# What Changes Have Occurred in the Pattern of Paediatric Burns in the Last Years with Special Attention to the COVID-19 Pandemic?

**DOI:** 10.3390/ebj4030032

**Published:** 2023-09-15

**Authors:** Sophie Y. Mok, Susan E. Adams, Andrew J. A. Holland

**Affiliations:** 1 Discipline of Paediatrics and Child Health, School of Clinical Medicine, Faculty of Medicine and Health, University of New South Wales, Sydney, NSW 2000, Australia; sophie.mok@student.unsw.edu.au (S.Y.M.); susan.adams@unsw.edu.au (S.E.A.); 2 Department of Paediatric Surgery, Sydney Children’s Hospital, Randwick, NSW 2031, Australia; 3 Injury Division, The George Institute for Global Health, Newtown, NSW 2042, Australia; 4 Department of Paediatric Surgery, Royal Alexandra Hospital for Children, Westmead, NSW 2145, Australia; 5 School of Paediatrics and Child Health, Sydney Medical School, University of Sydney, Camperdown, NSW 2006, Australia

**Keywords:** burns, child, aetiology, COVID-19

## Abstract

Burns in children remain a prominent mode of injury, resulting in considerable morbidity and mortality globally and are a key cause of disability-adjusted life-years. Paediatric burns present a unique challenge, in part due to the developmental, physical and emotional differences between adults and children. Those living in low- and middle-income settings are particularly vulnerable, facing problems such as overcrowding and floor-level cooking. During the COVID-19 pandemic, stay at home orders and the closure of schools and childcare changed the pattern of paediatric injury across the world, resulting in a general increase in trauma-related presentations. This review will examine recent global trends in paediatric burns, including the impact of COVID-19, specifically focusing on the pattern of burn aetiology over the past decade. It will also look at any changes with regard to epidemiological characteristics; burn site, severity and extent; first aid and location; and management and outcomes.

## 1. Introduction

Burns are responsible for considerable morbidity and mortality, affecting almost 9 million people globally each year [1]. Amongst children, they are the fifth most common cause of non-fatal injuries [2]. In this age group, burns present a unique challenge, in part due to the developmental, physical and emotional differences between adults and children. Mirroring trends in the adult population, children living in low-to-middle-income countries (LMIC) are particularly vulnerable to burns and face problems such as overcrowding and floor-level cooking [3,4]. Additionally, children in LMICs have limited access to prevention resources and treatment as a result of lower socioeconomic status. The pattern of burn injury changes over time, and it is worthwhile reviewing what changes have occurred in the past decade, especially with this previous decade including the COVID-19 era.

Times of significant social disruption place children at increased risk of injury, due to changes in their environment, activities and the level of supervision. The COVID-19 pandemic was a period of profound change across the world, with children spending much more time at home due to the closure of schools and stay at home orders. The pattern of paediatric injury during COVID-19 showed decreased presentations to emergency departments (EDs) but increased severity and proportion of trauma-related injuries [5]. These changes do not reflect the impact of COVID-19 as a disease itself, but rather the impact of the government responses and policy decisions made during the pandemic period.

This review will examine recent global trends in paediatric burns, including the impact of COVID-19, specifically focusing on the pattern of burn aetiology over the past decade. It will also look at any changes with regard to epidemiological characteristics; burn site, severity and extent; first aid and location; and management and outcomes.

## 2. Materials and Methods

Papers for this Literature Review were sourced from PubMed and Scopus searches using MeSH terms and Text Words such as ‘paediatric’, ‘burns’, ‘epidemiology’ and ‘patterns’. To narrow search results to specific topics (e.g., to focus on COVID-19), more specific search terms and citation chaining were utilised. Initial search results were limited to papers published within the last 10 years, to focus on recent trends in paediatric burns. In addition, to study longer term historical trends, subsequent searches looked at studies over a period of 25 years. Supplemented manual searches using PubMed and Google Scholar were performed. The titles and abstracts of papers were manually screened to focus on the aetiology and epidemiological characteristics of paediatric burns, as well as injury outcomes. In total, 94 studies were retrieved for this paper.

## 3. Results

### 3.1. Incidence

Children account for a significant portion of burn injuries globally, with data from 20 middle-income countries showing that persons under 18 comprise 42% of all burns cases [6]. In 2019, the Global Burden of Disease (GBD) Study estimated that for children under 15 years, there were 2.65 million new burns globally [6]. Between countries, child burn mortality can vary from 0 to 9.5 deaths per 100,000, and there remains a negative correlation between GDP and burn mortality, particularly evident in regions of Asia and Africa [7]. The global burden of injury has declined since 1990 [8]. This trend is reflected in the data for burns, with a decrease in the age standardised rates of years lived with disability (YLD) from 1990 to 2017 (−21% between 1990 and 2007 compared to −1.5% between 2007 and 2017) [9].

#### Impact of COVID-19

During the COVID-19 pandemic, stay at home orders with the closure of schools and childcare meant that children spent much more time at home. This is important because many childhood injuries generally, and burns specifically, occur in the home [10]. In the US, several studies reported statistically significant increases in incidence of paediatric burn cases during the COVID-19 pandemic period [11,12,13]. One US multi-institutional study found that six out of eight institutions showed a measurable increase in the presentation of burn injuries during the COVID-19 pandemics and a proportional increase in burns within the total population of paediatric trauma patients (n = 394, 6.7% of patients vs. n = 522, 7.8% of patients) [12]. Similarly, in a UK tertiary institution, the percentage of burns as a proportion of ED presentations almost doubled during COVID-19 lockdowns [10]. This was despite the total number of ED presentations decreasing by 60% (n = 7217 vs. 2936). Changes in incidence were not only seen in high-income countries (HICs), with a study from Morocco showing increased numbers of children admitted for paediatric burn injuries [14] and a study from Brazil reporting a significant increase in burns in children under 2-years old [15].

### 3.2. Burn Aetiology

Burns in children are broadly classified according to the heat source, including contact, flame, scald or other [16]. Information about the aetiology of burns is critical for guiding injury prevention. The mechanisms by which patients suffer from burns of different causes is different (e.g., touching an iron vs. spilling a cup of hot water) and different prevention initiatives may be required. In some cases, burn aetiology has likely implications on approximate injury depth and likelihood of operative treatment, such as in the case of flame burns [17].

#### 3.2.1. Scald Burns

Scalds remain the most common source of burns in children globally in both HIC and LMICs, accounting for the majority (53–76%) of paediatric burns in countries from Europe [18,19,20,21], the Americas [22,23,24,25], the Middle East [26,27,28,29], Asia [30,31,32], Australasia [17,33,34] and Africa [35,36]. A descriptive analysis using the World Health Organization’s Global Burn Registry (GBR) over a period of five years (2017–2021), suggests that scalds account for 62% of paediatric burns in middle-income countries [6]. However, the GBR cannot provide a comprehensive picture of burn trends in all middle-income countries, as participation is voluntary and only 20 countries were included in this analysis.

Some single country studies report higher proportions of burns are due to scalds. A 2014 review of 61,068 cases from China’s military hospitals, which treat 30% of the country’s paediatric burns, reported that 88% were due to scalds [37]. Similarly, two retrospective reviews from Eritrea and Hong Kong reported that scalds accounted for over 90% of all burn cases [38,39]. While both papers were limited to analysis of data from specialised burns units, they suggest how socio-cultural characteristics may influence the pattern of burn injury. For example, high-density living in Hong Kong increases the presence of hot liquids in common living spaces [39], and in Eritrea children eat meals on the floor next to the stove where liquids are boiled [40].

#### 3.2.2. Flame Burns

Flame burns are usually reported as the next most common, with estimates suggesting that they account for 18–29% of paediatric cases [18,21,26]. Paediatric flame burns are particularly common in LMICs [3,6], with the largest hospital in Sub-Saharan Africa confirming flame burns as the second most common cause after scalds [41]. In a number of African countries, such as Nigeria and Kenya, flame burns can be more common than scalds [42,43]. This may be partly attributed to the layout of many homes in these countries, where common areas are used for both sleeping and cooking with an open charcoal stove—a ‘Jiko’—on the floor.

#### 3.2.3. Contact Burns

In reports from HICs, paediatric contact burns are more common than flame—accounting for 13–30% of cases [20]. A 2011 prospective Australian cohort study found that 30.5% of burns were contact burns: this study was particularly important, as it included both inpatient and ambulatory patients and used specialised burns staff to correctly sort burns by aetiology [17]. The higher proportion of contact burns to flame burns in this region may be attributed to effective prevention strategies against flame burns established in HICs, including smoke detectors, safety cigarettes, decreased smoking, safety matches, safety cigarette lighters and induction cooktops [44].

#### 3.2.4. Regional Differences in Burn Aetiology

Within continents and countries, burn incidence, aetiology and burden can vary with socioeconomic status [45] and geographic location [46]. A study in South Central China revealed that, while there was no significant differences in burn aetiology between rural and urban paediatric patients, rural patients required more operative intervention, greater length of stay (LOS) and more expensive admissions [47]. A 2015 study of 4368 Australian children over 5 years revealed that children living in rural areas were more likely to sustain contact burns than scalds (40.8% contact, 37.7% scald) in comparison to metropolitan children (27.4% contact, 58.8% scald) [46]. Additionally, rural Aboriginal and Torres Strait Islander children, have almost double the rates of flame burns when compared to other Australian children (18% vs. 8%) [48]. The disproportionate burden in Indigenous populations is also reflected in a 2010 study from the American Burn Association’s National Burn Registry, which reported that Native American patients were younger than Caucasian patients and that in the 5–9 year age group, Native American children were almost 1.5x more likely to suffer flame burns than Caucasian children [49]. The rural/urban divide, however, is not universally reported [47].

#### 3.2.5. Impact of COVID-19

In HICs, there were changes recorded in the pattern of burn aetiology during the COVID-19 pandemic, with increases in scald, steam and flame burns widely reported. The Australian state of Victoria was the most locked down region in the world—a cumulative 262 days with strict restrictions such as overnight curfews and travel limits [50]. Their statewide burns service reported that during the 2020 lockdown period, the proportion of paediatric burns due to scalds or flames increased, compared to previous years [51]. Similarly, an increased proportion of scalds (68% to 85%) from a pre-COVID-19 cohort to a COVID-19 cohort was also documented in the UK [10]. Another UK tertiary institution theorised that food-and-drink-related thermal burns would increase with more baking and meal preparation during lockdown; however, no such increase was shown [52]. A study based in an Italian children’s hospital showed that hot liquid burns accounted for almost all burns (97%) but did not compare to a pre-COVID cohort and contained a small sample size (n = 47) [53].

A large increase in steam-inhalation scald burns was noted during the height of the pandemic. In England, a survey of 86% of paediatric burn services described a 50% increase in this scald mechanism. This likely reflected the increased use of steam inhalation as a home remedy to treat COVID-19 [54]. Similarly, a US-based study reviewing patients in the Burn Care Quality Platform Registry reported a 200% increase in head and neck burns, with scalds being the most common cause of burns. The authors also postulated that steam inhalation contributed to this burden of disease [55]. 

In the US, an increase in overall numbers of burns was reported, with flame burns accounting for a higher proportion than pre-pandemic. A multi-institutional study including 916 in-patients from eight Paediatric Trauma Centres in the US comparing a pre-COVID-19 control cohort (n = 394) with a COVID-19 cohort (n = 522) found that, in addition to higher numbers of burn injuries admitted to hospital, there was also variation in the relative types of burns sustained. The proportion of flame burns increased (19.0% to 26.8%) whilst contact burns decreased (28.4% to 22.3%) [12]. Similarly, a retrospective single-centre study of a tertiary paediatric hospital in the US compared the ED presentations of COVID-19 (n = 392) and pre-COVID-19 cohorts (mean n = 467.6), to find an increase in flame burns from 22.4% to 32% (*p* < 0.001) [13].

There were also changes seen in the aetiology of paediatric burns in LMICs. A study of Brazilian Burn Treatment Centres described an increase in chemical burns due to alcohol across all age groups, including children [56]. This may be related to the common cultural practice in Brazil of using alcohol for cleaning and the COVID-19 prevention campaigns which encouraged the use of alcohol as an antiseptic cleaning product. Contrastingly, a Brazilian Trauma Unit reported no significant changes in cause of burns in both adults and children [57]. A children’s hospital in Turkey reported that 74.2% of paediatric burn admissions were for scalds but did not compare to a pre-COVID cohort to demonstrate change [58].

During the COVID-19 pandemic, one study recorded no increase in the presentation of burns caused by non-accidental injury (NAI) [59], but overall there was limited research into the incidence of NAI via burns during the pandemic. Additionally, both increases and decreases of NAI were reported during the COVID-19 pandemic [60]. 

### 3.3. Age

The age distribution for paediatric burns is almost always bimodal with peaks in pre-school and teenage years, and this has not changed in the last 10 years. Children under the age of 5 are most susceptible [61], with 1–5 year olds accounting for up to 61–73% of paediatric burns [6,18,19,21]. In this age group, scalds are the primary mode of burn for up to 80% of cases [6,24,62]. This may be due to the developmental stage of young children, where they develop the motor skills to explore their environments but lack self-awareness, hazard perception and the ability to assess risk. In contrast, flames are the most common mechanism of burn for teenagers, likely due to risky behaviours and increased exposure to occupational hazards [17,22,37]. Particularly in HICs such as Australia, flame burns are uncommon in young children and more prevalent in older children and adolescents (15.4%) [34]. A 2020 Australian study of only school-aged children (4–18 years old) found that flame burns (31%) closely followed scalds (33%) in frequency [16].

#### Impact of COVID-19

During the COVID-19 pandemic, a Women and Children’s Hospital in the UK reported an increase in mean age of burn patients from 2.9 years in a 2019 control period to 4.8 years in a 2020 COVID-19 lockdown period [10]. They proposed the shift to be related to older children spending less time at school and more time at home, where most burn injuries occur. Similarly, research performed at a level 1 trauma centre in Israel found that while 0–2 year-old children were at greatest risk of burns in 2017 and 2019, 2–5 year-old children were at the greatest risk of burns during the COVID-19 pandemic, with 89% of cases occurring during cooking or dining [63]. Other studies also describe similar age groups to be most at risk, with a Turkish study reporting a mean age of 4.5 years [58] and an Italian study reporting most burn injuries occurring in the 0–4 group [53].

### 3.4. Sex

Males predominate in all age groups for burns. It is generally believed that this can be attributed to differences between male and female perception and assessment of risk, together with likelihood of involvement in dangerous behaviours. The higher proportion of males in paediatric burn injury is consistent across the world—both in HICs [18,22,61] and LMICs [6,18,22,31,61]. Some European studies have also found that the ratio of male to female patients increases as age increases, potentially up to 4:1 above the age of 10 [21] or 5:1 above the age of 15 [18]. Contrastingly in South Asia, a systematic review of 40 years of papers on burns in India, Pakistan, Bangladesh and Sri Lanka noted that while males still account for the majority of burns, in adolescence the burden of injury increases for females, as girls shift toward contributing to domestic tasks such as cooking and wear loose fitting clothes [64].

#### Impact of COVID-19

The effect on the ratio of burns in males vs. females during the COVID-19 pandemic was not widely explored [51]. One study of a level 1 trauma centre in Israel uniquely noted that in comparison to a pre-COVID period, the proportion of females increased from 43% to 75% during the COVID-19 period [63]. Additionally, all burns to female children during this time were scalds, suggesting that this increase in burns to female children may have been due to greater time spent unsupervised in the home, including preparing food and drinks, while unsupervised boys may have been inclined to more risk-taking behaviour, known to be correlated with flame burn.

### 3.5. Total Body Surface Area

Total body surface area (TBSA) refers to the percentage of the body’s surface that is affected by the burn. Ranges differ between studies, reflecting the variation between EDs receiving smaller burns and specialised burns units targeted at managing severe burns. While it is seldom completely accurate [19], calculating TBSA does aid treatment decisions, and is a predictor for mortality [32] and duration of admission [28,65]. In children, burns that affect <10% of TBSA are most common and usually account for 61–75% of cases [18,21,37] and up to 90% in some cases [33,39]. The aetiology of the burn is related to the TBSA. Contact burns and scalds are more commonly less than 10% TBSA [17,18], whereas flame burns are more likely to be >10%, especially those from explosions and misuse of fire [18].

#### Impact of COVID-19

Burns were reported to be larger and more severe during the COVID-19 pandemic [10,15,53]. At a UK tertiary hospital during the COVID-19 pandemic, the proportion of patients with >5% TBSA burns increased, accounting for half of all paediatric burns [10]. This suggests that government pandemic restrictions altered perceptions about the level of injury severity that warranted a hospital visit and may also reflect a change in management patterns due to health system pressures. Also in Europe, an Italian study of 47 patients reported that mean TBSA was 14 ± 11% [53]. In LMICs, a Turkish study reported mean TBSA to be 12% [58], and a Brazilian study found that there was a statistically significant increase in both median TBSA and the number of children with full-thickness burns between a 2019 cohort and 2020 COVID-19 cohort [15].

### 3.6. Body Area Affected

The commonest area of the body affected by burns varies widely in published studies over the last 10 years, with most body regions claiming highest prevalence depending on the study design, setting and age group studied. As the aetiology of burns varies according to body region, this in turn affects resource allocation and injury prevention initiatives.

Hands are a common body area affected by burns, reportedly the commonest [34], especially in children under the age of 5 years [61]. US and Australian papers have found that contact burns are a common source of hand burns (61.4–75.5%) [17,66], with the majority occurring on the palmar aspect of the hand [67,68] affecting either the palm only or the fingers [69]. Hand burns are particularly common in children, with toddlers being at a high risk of sustaining palmar contact burns from touching hot objects out of curiosity and lack of caution [70]. Two studies in HICs found that the most common aetiology of hand burns was contact with a hot stove or oven [67,69], and fireplaces, irons and campfires are other common causes [67]. Friction burns from exercise treadmills are also a common mode of hand injury, accounting for 25% of hand burns over a 5 year period at a US level 1 paediatric burn centre and primarily occurring in the 0–4 age group [66]. In an LMIC, a 3-year study at a Bulgarian burns and plastic surgery clinic concluded that in the most common aetiology of hand burns was contact burns for children under 5 and electrical and flame burns for children over 5 [71]. This pattern of hand injury informs injury prevention initiatives, which need to focus on preventing young children from being unsupervised around dangerous objects (e.g., hair irons, treadmills) and keeping these objects out of reach [67].

#### Impact of COVID-19

The majority of studies surrounding paediatric burns during COVID-19 do not mention patterns in the body area affected by burn injury. A French Burns Unit observed statistically significant increases in hand and lower limb burns in a COVID-19 population [72], while one Israeli study noted that the upper limb was the most common body part injured, with no change during the pandemic [63].

### 3.7. Location

The location that a child is most likely to be burned at is at home [38,39,61]. This is because of the amount of time children spend at home, as well as the hazards that are present in the kitchen and living areas, which are not configured with the primary aim of child safety. Within the home, the kitchen is a particularly dangerous location, accounting for most burns in numerous countries [64]. 

Home environments are typically more dangerous for children living in LMICs, as there is poorer implementation of preventative measures compared to HICs (e.g., smoke detectors, stove guards, fire screens) [35,73,74]. For example, in South Africa, informal settlements often house socioeconomically disadvantaged families and contain numerous cooking and heating hazards (e.g., paraffin stoves and exposed electrical wiring) [73]. These informal settlements are the riskiest home environments for children in South Africa, as identified by a 6-month retrospective study at Cape Town’s Red Cross Children’s Hospital [74]. In other LMICs such as Eritrea, children often play in combined cooking and living spaces, where they are at increased risk of contact with hot fluids and solids [38].

#### Impact of COVID-19

Several studies reported that during the COVID-19 pandemic, the proportion of burn injuries sustained at home increased and that, indeed, burns at home accounted for most burn presentations [10,57,75]. This reflected the increased amount of time, which children spent at home during the lockdown period with the closure of childcare and schools. Additionally, some studies noted that children often remained unsupervised at home during this time, as parents were required to work from home [75]. While some studies cite lack of adult supervision at time of injury as a preventable risk factor [76], the presence of an adult does not eradicate the risk of a child burning themselves [35]. 

In contrast, some studies showed no changes to proportion of paediatric burns that occurred in the home, with an Australian study suggesting that this may be due to a saturation effect where the majority of burns already occur at home [51].

### 3.8. Management and Outcomes

#### 3.8.1. First Aid

Burn first aid alleviates pain, reduces the depth of burn injury and decreases healing time [77]. Some physical characteristics, such as thinner skin, predispose children to deeper burns and increase the importance of first aid for paediatric burn injury [70]. Gold-standard first aid involves running cool water over the injury site for at least 20 min, within the first three hours post-burn whilst avoiding hypothermia [68,77]. From a public health perspective, there is a need to enhance education about the importance of immediate first aid before hospital presentation.

Despite its importance, the administration of first aid remains inconsistently documented, with most studies failing to mention first aid at all, and even if it is recorded there appears limited reported data between the different types of first aid and documentation of its duration. 

Where it is studied, first aid is often reported to be suboptimal. A 2014 Australian study found that 66% of patients either received no first aid or non-gold-standard first aid, with this high rate possibly reflecting the paper’s unique inclusion of both inpatients and outpatients [34]. This had improved somewhat by 2020, where an Australia and New Zealand study found the lack of first aid almost halved to 34% (16% receiving none and 18% receiving suboptimal) [16]. This indicates that public health campaigns between 2014 and 2020 were effective but have some way to go in achieving sustained universal first aid at the scene. Outside of HICS, first aid continues to be inconsistently administered and recorded [39,47]. A tertiary institution in South Africa recorded that 63% of patients received some first aid, although type was not recorded [41]. A tertiary institution in Nigeria reported that 75% of patients received some first aid but described poor administration of gold-standard first aid, with the most common treatment being raw eggs in 31% [35]. Across the world, knowledge about correct first aid is severely lacking. Even if individuals know to use running water, only 8–12% are aware of the correct duration of administration [78]. There is the added concern that running water is not available to all, suggesting that public health campaigns tailored to the environment are needed.

#### 3.8.2. Medical Care

Most burns in children are cared for in an ambulatory setting [25,79]. This has increased with a reduced need for daily dressings and changing models of care in HIC [80]. Inpatient care tends to be required for burns of greater severity, as indicated by a greater proportion of those admitted having larger TBSA than outpatients [25]. For inpatient care, mean LOS varies from 7.4 to 11.5 days [6,20,22,37]. This reflects the diverse range of burn severity across papers and differences in models of health care funding. For example, in the US, LOS tends to be shorter, perhaps related to differences in healthcare funding compared with other HICs. A retrospective 2003–2016 analysis of the Kids’ Inpatient Database, which collects data from burn centres across the US, recorded median LOS as 1.9 days [81]. In countries with a more socialist medical model, LOS is typically much longer. For example, a retrospective 2013–2019 review conducted at the largest burn centre in Central China recorded that the median LOS was 15 days [82]. Certain groups of children are more likely to sustain severe burns, or live further from the treating hospital and require longer LOS, including children from a lower socioeconomic background [19,83], living in rural areas [46,47] or from an Indigenous background [48].

The proportion of patients who are admitted to an intensive care unit (ICU) and require mechanical ventilation varies depending on the type of facility and the demographic of patients who present there. In an HIC, rates of ICU admission may be as low as 4%, with 65% of those patients requiring mechanical ventilation [33]. Alternatively, in LMICs such as Turkey, it has been reported by a single tertiary institution that a quarter of patients are admitted to the ICU and that this rate increases as age increases [84]. 

Operative intervention is required in a large proportion of paediatric burn cases and typically involves surgical debridement and sometimes skin grafting [16]. There is international variation, with operative intervention required in 40% of cases in China [47] and Malawi [85], compared to 68% of cases in Australia [33]. Flame burns require the most operative intervention (38%), followed by scald (17%) and contact burns (12%) [17]. It should be noted that there is inconsistency between studies for what is defined as operative intervention, with some studies only focusing on grafting, while others also include debridement sessions.

In HICs, the rates of in-hospital mortality are low and are usually below 2% [16,47,84]. There have been improvements in mortality over time, with analysis of a US paediatric inpatient database (n = 39,443) showing that, from 2003 to 2012, there was a reduction in mortality rate by 48.1% and hospitalisations by 4.6% [81]. However, patients in LMICs have worse outcomes than patients in HICs [35], with an 8-year-long retrospective study showing that, in comparison to North American patients, Dominican patients had higher rates of mortality, as well as larger TBSA and longer LOS [86]. In LICs, child mortality from fire and flames is also nearly 11 times higher than in HIC [87]. In general, flame burns are associated with higher levels of mortality [28], with a study in Malawi finding that, after controlling for other factors (such as age, sex and TBSA), in-hospital mortality from flame burns was 2.6 times the mortality of scald burns [85]. Mortality is also positively correlated with TBSA [82]. Burns of >40% TBSA [88] and >70% TBSA [28] have been found to be associated with a high mortality and morbidity rate.

#### 3.8.3. Impact of COVID-19

During the COVID-19 period, there was limited documentation of the administration of first aid for paediatric burns, so no change can be noted. Medical care adapted across the world in response to government-enforced policy, with lockdowns limiting face-to-face consultations, substituted by telemedicine. The lack of immediate contact and the poor video image quality posed an issue for the accurate assessment of depth and wound condition, the expertise of burn dressings, and the long-term management [89]. 

In terms of the pattern of inpatient paediatric hospital admission during COVID-19, generally there were fewer inpatient admissions and shorter LOS reported. A UK women and children’s hospital reported reductions in in-person clinical reviews by 61%, the number of inpatients by 37% and LOS by 31% [10]. In the US, a multi-institutional study found reduced rates of inpatient hospital admissions but unchanged LOS, as well as unchanged rates of ICU admission but increased ICU LOS [12]. A study from the only Romanian Paediatric Burns Department also showed a decrease in inpatient admissions by 41% [90]. The authors theorised that the reduction was due to fewer admissions of minor burns (< 10%), as the number of burns with TBSA 10–80% was unchanged between the pre-COVID and COVID period. In Israel, the ED of a level 1 trauma centre reported no statistically significant change in the rate of hospitalisation [63], while the ED of a paediatric tertiary institution reported an increase (4.5% vs. 2–2.6%), as well as longer duration between injury and presentation. The LOS or rate of operative intervention was unchanged [91]. The latter study also described the adaptations of their hospital to improve triage during COVID-19, including an additional outpatient clinic and use of telemedicine. One hospital in Melbourne, Australia, reported that mean LOS increased from 1–5 days pre-COVID-19 to 2–16 days during COVID-19, theorising that the longer LOS was a result of higher burn severity during lockdown [51].

### 3.9. Limitations

Accurate analysis, interpretation and comparison of literature reporting burns in children remains problematic because of the varying study locations, cohorts, as well as inclusion and exclusion criteria. Firstly, studies in individual countries may be conducted in a variety of locations, ranging from highly resourced, specialised burns units to general paediatric surgery units, EDs and outpatient clinics. Additionally, the only results that can be analysed are from centres that are resourced to conduct and publish research, potentially biasing results. 

Secondly, there is no universal agreement on the definition of a ‘paediatric patient’. This can be attributed to cultural, legal and funding differences between health systems and institutions, resulting in inconsistencies between studies and an inability to compare equivalent data. This means that a ‘paediatric burn patient’ may only include children under the age of 13 in some countries [36], while the age may be extended to 20 in others [31,62]. Even within Europe, the majority of countries define a paediatric patient as under the age of 18, but some countries limit the age to 14 or extend it to 19 [92]. It follows that studies that exclude older children may report higher proportions of scalds, which are more prevalent in the young [6,24,62].

Thirdly, while the majority of burn presentations do not involve inpatient admission, most studies only comprehensively analyse inpatients, ignoring the large subset of patients who are managed in an ambulatory setting [25,34,45,79]. This represents an important limitation, as many burns in children, especially in HICs, will involve lower total body surface areas (TBSA) and are almost exclusively treated in an ambulatory care setting [79]. Australian-based studies have found that contact burns account for more outpatient than inpatient cases (44.5% vs. 27.1% [34]; 39.2% vs. 24.4% [79]) while the opposite is true for flame burns (2.9% vs. 11.5% [34]; 3% vs. 21.8% [79]). It is important for future burn registries and studies to include outpatient data because of the epidemiological and aetiological differences between inpatient and outpatient populations, as well as to match increasing use of ambulatory care for burns management, which is more affordable [19,93].

Finally, there is a need for consistent categorisation of burn injury aetiology. While most papers use the categories based on the source of tissue damage (scald, flame, contact, electrical, and other), some papers use more descriptive terms that cross categories. For example, one paper cites “contact with heat and other substances, hot drinks, food, fats and cooking oils” as the most common cause of burns in children, a category which includes both scalds and contact burns [61]. Knowledge about burn aetiology is critical when designing prevention programs. There is also limited study of the efficacy of prevention programs, with a small number of papers solely focusing on prevention programs [94]. Overall, there is a need for further study in this area to adapt and improve prevention programs.

## 4. Conclusions

Burns remain a prominent health issue for children across the world. The COVID-19 pandemic brought significant change to the lives of children and their risk of trauma-related injuries such as burns. This has implications for the public health response to future social disruptions. Changes in the aetiology and epidemiology of paediatric burns should be consistently analysed in updated literature with a view to providing direction for future public health education and prevention programs in all environments in which children live. 

## Data Availability

Data sharing not applicable.
